# Conserved HIV Epitopes for an Effective HIV Vaccine

**DOI:** 10.4172/2155-9899.1000518

**Published:** 2017-08-30

**Authors:** Bikash Sahay, Cuong Q. Nguyen, Janet K. Yamamoto

**Affiliations:** Department of Infectious Diseases and Immunology, College of Veterinary Medicine, University of Florida, P.O. Box 110880, Gainesville, FL 32611-0880, USA

**Keywords:** Vaccine epitopes, B-cell epitopes, T-cell epitopes, HIV vaccine

## Abstract

Despite major advances in antiretroviral therapy against HIV-1, an effective HIV vaccine is urgently required to reduce the number of new cases of HIV infections in the world. Vaccines are the ultimate tool in the medical arsenal to control and prevent the spread of infectious diseases such as HIV/AIDS. Several failed phase-IIb to –III clinical vaccine trials against HIV-1 in the past generated a plethora of information that could be used for better designing of an effective HIV vaccine in the future. Most of the tested vaccine candidates produced strong humoral responses against the HIV proteins; however, failed to protect due to: 1) the low levels and the narrow breadth of the HIV-1 neutralizing antibodies and the HIV-specific antibody-dependent Fc-mediated effector activities, 2) the low levels and the poor quality of the anti-HIV T-cell responses, and 3) the excessive responses to immunodominant non-protective HIV epitopes, which in some cases blocked the protective immunity and/or enhanced HIV infection. The B-cell epitopes on HIV for producing broadly neutralizing antibodies (bNAbs) against HIV have been extensively characterized, and the next step is to develop bNAb epitope immunogen for HIV vaccine. The bNAb epitopes are often conformational epitopes and therefore more difficult to construct as vaccine immunogen and likely to include immunodominant non-protective HIV epitopes. In comparison, T-cell epitopes are short linear peptides which are easier to construct into vaccine immunogen free of immunodominant non-protective epitopes. However, its difficulty lies in identifying the T-cell epitopes conserved among HIV subtypes and induce long-lasting, potent polyfunctional T-cell and cytotoxic T lymphocyte (CTL) activities against HIV. In addition, these protective T-cell epitopes must be recognized by the HLA prevalent in the country(s) targeted for the vaccine trial. In conclusion, extending from the findings from previous vaccine trials, future vaccines should combine both T- and B-cell epitopes as vaccine immunogen to induce multitude of broad and potent immune effector activities required for sterilizing protection against global HIV subtypes.

## Introduction

In an USAID global survey, approximately two million new HIV-1 cases were reported in 2015 alone, despite the increased use of the antiretroviral therapies (ART) to control the global spread of the infection [1]. Daily administration of ART successfully lowered circulating HIV load to minimal to undetectable levels in HIV-positive (HIV+) individuals and prolonged their life to nearly normal lifespan [2]. In 2015, approximately 17 million of 37 million (46%) HIV+ individuals received ART, incurring tremendous toll on their country’s economy and on the global community [1,3]. To further minimize the global spread of the viral infection, ART must be provided to rest 54% of infected individuals including millions of HIV-negative high-risk groups as pre-exposure prophylaxis [3]. In 2016, UNAIDS and WHO projected approximately 33 million HIV+ individuals would be on ART by 2030, which would lead to a ten-fold decrease (<200,000 individuals) in newly infected individuals and a two-fold decrease (<400,000 individuals) in individuals succumbing to the HIV infection [1,3]. Although the rise in the drug resistance has so far been modest, there is a small portion of cases with high drug resistance in ART-naïve HIV+ individuals in certain countries; additionally, resistance also seen in individuals restarting the ART after treatment interruptions [3]. More importantly, the long-term use of the best drug combinations of ART is still unable to cure HIV infection [2]. The cost of treatment, potential drug resistance and inefficacy of the existing treatment underscore the urgent need to develop an effective prophylactic vaccine against HIV-1.

The current review will describe the findings important to the development of an effective HIV vaccine with emphasis on B-cell and T-cell vaccine epitopes through the coverage of the following topics: 1) Past and present understanding of HIV vaccination, 2) Conserved B-cell and T-cell epitopes on HIV, 3) B-cell epitopes generating broadly neutralizing antibodies (bNAbs), 4) Non–neutralizing antibodies (nAbs) during HIV vaccination and infection, 5) Conserved T-cell epitopes associated with anti-HIV activity(s), 6) Conserved T-cell epitopes associated with protective HLA allotypes prevanlent in HIV endemic countries, 7) Antibodies and T-cell responses enhancing HIV infection, and 8) Conserved T-cell and B-cell epitopes with potent anti-HIV activity for HIV vaccine.

## Past and present understanding of HIV vaccination

Vaccination changed the conventional disease management; an effective vaccine can reduce or even eradicate the infectious disease, which made vaccines as an essential tool against infectious agents. Several unsuccessful attempts made in the past in the development of such vaccines provided extensive information that can be used in the future for more effective vaccines against the virus. Here we reviewed several vaccination attempts and their cause of failures to protect against HIV-1. Since the initiation of the first phase-III clinical vaccine trial (VAX004) in 1998, three independent phase-III clinical vaccine trials ended with marginal to no efficacy (Table 1) [4–6]. All of these trials tested HIV-1 vaccines containing viral envelope (Env) protein(s) [4,5] or its combination with viral Gag (matrix-p24-nucleoprotein) and protease (Pro) proteins [6]. The overarching goal of these trials was to induce HIV-1 neutralizing antibodies (NAbs) in the vaccinated subjects and to evaluate their efficacy [4–6]. Additionally, the last trial RV144 focused on inducing both humoral and cellular immunity against HIV-1, especially by generating broadly NAbs (bNAbs) using a prime-boost system with vectored gag-pro-env prime and boosting with Env proteins from two subtypes or clades [6]. Unfortunately, these trials were unsuccessful in inducing bNAbs against global HIV subtypes; however, the last trial conferred a modest prophylactic protection [4–6]. Nevertheless, these trials demonstrated the production of HIV-1 type (tier 1)-specific NAbs and substantial levels of Env-binding antibodies (Table 1).

The RV144 trial was minimally successful by conferring 31.2% protection in the combined low/medium/high-risk group but conferring only 3.7% protection in the high-risk group [6]. Since the type-specific NAbs did not correlate with the modest protection observed in the combined group, other immune correlates of protection were evaluated, such as non-neutralizing anti-HIV antibodies (nNAbs) and T-cell immunity [7]. Notably, the presence of Env-specific IgG nNAbs inversely correlated with HIV infection with a positive correlation with the protection [7–9]. However, Env-specific nNAbs with antibody-dependent cellular cytotoxicity (ADCC) activity and Env-specific CD4+ T cells directly correlated with the protection from active infection [7]. Conversely, Env-specific IgA antibodies positively correlated with HIV infection (i.e., inverse correlation with protection) [7]. A recent report showed IgA antibodies produced in RV144 trials inhibited the protective ADCC activity [10]. This observation raised concern since mucosal IgA immunity is considered more or equally important as IgG immunity against mucosal transmission of HIV infection, which is the major transmission route for HIV [11].

Prior to the completion of RV144 trial, two phase-IIb clinical vaccine trials (Step and Phambili trials) consisting of adenovirus type-5 (Ad5) vectored HIV gag/pol/nef vaccine were in progress and tested whether vaccines based solely on anti-HIV T-cell immunity could confer protection in vaccinated subjects (Table 1) [12–14]. Ad5-gag/pol/nef vaccination did not induce NAbs, bNAbs, or ADCC nNAbs to Env due to the absence of HIV env gene in the construct. Unfortunately, upon one-year evaluation, the vaccine group showed enhanced HIV infection compared to the placebo group in the Step trial, resulting in an abrupt termination of both Step and Phambili trials. Some reasons for the failure of the Step trial were attributed to: 1) the pre-existing anti-adenovirus antibodies in the enrolled subjects [12,15], 2) the induction of Ad5-specific CD4+ T cells with increased susceptibility to HIV and/or increased HIV trafficking to mucosal sites [12,15–18], 3) more uncircumcised subjects in the vaccinated group than in the placebo group [12,15], despite previous studies showed circumcision directly correlating with decreased HIV transmission [19,20], 4) the enhancement of HIV infection caused by the non-specific release of IFNγ [21], and 5) the poor induction of anti-HIV T-cell immunity [22,23]. In fact, the anti-HIV CD8+ T-cell responses were of low magnitude and narrow breadth, less polyfunctional, and targeted predominantly Pol and Nef proteins instead of Gag protein. CD8+ cytotoxic T lymphocyte (CTL) and other T-cell responses against HIV Gag are associated with control of HIV infection compared to the T cell responses directed against Pol [24–29]. Hence, insufficient magnitude and quality of anti-HIV T-cell immunity were induced to counteract the HIV enhancing effects caused by adenovirus vector and experimental design of the Step trial that might have caused the failure of the trial. The initial nine-month results of the Phambili trial did not show any statistical change in the rate of infection; however, it was difficult to draw any conclusion due to early termination and low participation compared to the Step trial [13]. The findings from this trial indicated that neither pre-existing Ad5 titers nor circumcision status affected the vaccine efficacy, which was later confirmed in the median 42-months follow-up analysis [14]. Moreover, the follow-up analysis revealed more infection in the vaccine group than placebo group leading to the conclusion that vaccination significantly increased the risk of HIV infection.

Since none of the vaccines in the Phase-III vaccine trials induced potent bNAbs, scientific efforts were subsequently focused on identifying the B-cell epitopes that induce potent bNAbs against global HIV subtypes [30–32]. The findings from RV144 also sparked renewed interests in identifying B-cell epitopes on HIV Env that induced anti-HIV nNAbs (e.g., ADCC Abs) as well as on identifying protective conserved HIV T-cell epitopes to be combined with protective B-cell epitopes as immunogens for an effective HIV vaccine [33–35]. The recent findings about bNAbs as discussed below further support the need for combining B-cell epitopes for ADCC nNAbs and bNAbs/NAbs with anti-HIV T-cell epitopes [22,34–36].

## Conserved B-cell and T-cell epitopes on HIV

An epitope is defined as the site on a molecule or an antigen recognized by either an antibody or B-cell receptor (BCR), or by T-cell receptor (TCR) recognizing the antigenic peptide bound to the major histocompatibility complex (MHC) [37–39]. Hence, a B-cell epitope binds to the epitope-specific antibody as well as epitope-specific BCR on B cells. The binding of the antigenic epitope to the BCR(s) triggers the B-cell differentiation into epitope-specific antibody producing clonal B cells. A B-cell epitope can be either continuous (linear) or discontinuous (conformational) stretch of amino acids (aa) on the antigen [40]. Continuous or linear epitope consists of a consecutive aa sequence on the antigen. Whereas, a discontinuous or conformational epitope is formed with the help of more than one continuous aa series come in close vicinity due to protein folding and other proteinaceous and non-proteinaceous interactions. B-cell epitopes on HIV can also be found on lipid, glycan, and protein antigens or their combination (e.g., lipoprotein, glycoprotein) on HIV [30,31,34,41].

In recent studies, a majority of bNAbs reacted to conformational epitopes than to linear epitopes (Table 2) [30,31,34,42] making it difficult to use them in vaccine development where isolated epitopes are used in vaccines. HIV epitopes for bNAbs are described conserved because of their cross-reactivity due to broader affinity to multiple HIV subtypes [34,43]. However, not all bNAb contact site of the HIV epitopes are highly conserved in their aa sequence but can include highly variable segment(s), suggesting other than aa sequences controlling their binding. The binding of bNAb without a common aa sequence may be due to the common conformation of the epitopes despite variation in aa sequences or protein modifications (glycosylation, lipidation) that might be the cause of their binding that cannot be replicated in a vaccine with ease.

In comparison, T-cell epitopes are short linear peptides processed from a protein antigen, and presented on MHC molecules for their recognition by TCR on T cell to induce its effector function(s). The conserved T-cell epitopes on HIV are identical or similar (homologous) in aa sequences and are conserved among the HIV isolates from the same subtype (type-specific) or from multiple HIV subtypes [44–46], and those evolutionarily preserved are conserved among AIDS lentiviruses of humans, nonhuman primates (NHPs), and cats (HIV, SIV, FIV) [47,48]. Generally, those conserved among HIV/SIV/FIV are often conserved within and among HIV subtypes [47,48]. The evolutionarily conserved epitopes that maintained their existence among different hosts have lower likelihood to acquire mutation(s) compared to the non-conserved epitopes with variable aa sequences. It is generally perceived that the highly conserved epitopes are present on protein regions essential for viral survival, and any substantial mutation(s) would affect the fitness of the virus [49–51]. Immunization with conserved HIV T-cell epitopes can have diverse outcomes, ranging from infection-enhancing, neutral, beneficial, or protective effect. Therefore, careful selection of conserved T-cell epitopes that induce potent anti-HIV immunity is needed to develop a highly effective HIV vaccine against global HIV subtypes.

## B-cell epitopes generating broadly neutralizing antibodies (bNAbs)

The bNAbs are those anti-HIV antibodies that potently neutralize a broad spectrum of heterologous HIV viruses including those among global HIV subtypes [34,43]. The existence of bNAbs has been first detected in 20–50% HIV+ individuals with chronically infected for over 2–5 years [34,52,53]. According to the studies with bNAbs isolated from infected subjects, bNAbs target five epitopic regions of HIV Env [30,31,34] and these include (Table 2): 1) CD4-binding site (CD4bs), 2) V2 proteoglycan moiety on the trimer apex of surface Envs (SUs), 3) V3 proteoglycan moiety on the high mannose patch of SU, 4) membrane proximal external region (MPER) of Env transmembrane domain, and 5) gp120-gp41 interface with or without fusion protein. HIV antigenic epitope(s) that induce bNAbs with above characteristics should be considered an ideal vaccine antigen(s) for prophylaxis. The gp120-gp41 interface epitopes are often transitional epitopes requiring initial contact to CD4 and/or CCR5 (co-receptor) molecules or requiring viral fusion process before they are sufficiently exposed to the bNAbs [54]. Since the majority of the known human bNAbs have been IgG isotypes (Table 2), such vaccine epitopes should at least induce IgG bNAbs. The results from RV144 suggest that elevated levels of IgG1 and IgG3 subclasses correlated with vaccine protection, whereas high levels of IgA and IgG4 correlated with enhanced HIV viral titer [9,10,55]. As of to date, the HIV vaccine epitopes for bNAb have not been identified or developed [31,34,56,57]. Vaccines consisting of recombinant Env and trimeric Env proteins have not successfully induced bNAbs [4,5,58,59]. However, information about the characteristics of the bNAbs has been described as the first step towards developing their counterpart Env epitopic antigen or immunogen for vaccine [30,31,34].

The antigen binding site on an antibody that recognizes an antigenic epitope is called paratope of the antibody [37]. Antibodies stimulated by an antigen upon immunization or infection possess different paratopes that bind to different epitopes on an antigen. The paratope of an antibody is found on complementarity determining regions 1, 2, and 3 (CDR1, CDR2, CDR3) of the antibody heavy and light chains. The characteristics of the bNAb paratopes derived from chronically HIV-infected subjects have recently been described [30,31,34,60]. The bNAbs with 50% breadth develop in 20–50% of chronically HIV-infected subjects [52]. The bNAb paratopes have unique characteristics [30,31,34,61,62] such as: 1) high levels of somatic hypermutations at theV(D)J antibody genes, 2) often possessing long heavy-chain CDR3 (HCDR3), 3) often showing polyreactivity and autoreactivity with self-protein, glycan, and lipid, and 4) taking years to develop the broad specificity of bNAb paratopes.

Although few occluded bNAb-inducing Env epitopes (e.g., CD4bs) may remain relatively constant in aa sequence, mutation(s) at other viral Env site (e.g., glycan insertion or deletion) of the viral escape variant(s) may expose the occluded bNAb epitopes [63,64]. For example, bNAbs developed to V2 proteoglycan moiety may lead to viral escape variant(s) with N167D which in turn exposes the occluded CD4bs resulting in the development of bNAbs to CD4bs. Such event can explain why a sizable number of chronically infected individuals possess multiple bNAbs with specificity to different targeted bNAb epitope region [64–67]. Another possibility is that the ancestral Env epitope of potential bNAb itself may need to undergo changes such as mutations with subtle conformational changes as part of viral escape from NAbs or viral co-evolution [63,64,68]. The latter scenario of co-evolution of both virus and B cells takes many years to develop bNAbs. In either scenario, affinity maturation of the bNAb B-cell lineages in response to viral escape variants may induce multiple cycles of somatic hypermutation in the antibody genes [63,69,70].

Many bNAbs were determined to be polyreactive and autoreactive to self-antigen(s) [61]. These observations raised a concern that immunization with vaccine antigen(s) consisting of bNAb epitopes may induce autoreactive antibodies, which may cause autoimmune disease in the vaccinated hosts. However, initial passive transfer of autoreactive bNAbs in nonhuman primates (NHPs) was well tolerated, and no sign of autoimmune symptoms was observed in the passively immunized monkeys [71]. Furthermore, the difficulty in developing bNAbs in chronically infected individuals has been attributed to the development of tolerance to the bNAb epitopes that are recognized as self-epitopes by the host immune system [61]. This view is based on a well-established immunological concept that autoreactive B cells often undergo clonal deletion and/or anergy to prevent the production of autoreactive antibodies in a healthy individual [61,72]. Besides these undesirable features (i.e., autoimmune and tolerance) to overcome, high levels of somatic hypermutation of antibody genes are required to develop bNAbs with or without long HCDR3. Thus, these combinations of events needed for developing bNAbs raised a major concern on whether bNAbs can be developed by vaccination [33,73].

## Non-neutralizing antibodies (nNAbs) during HIV vaccination and infection

Some nNAbs and many of the bNAbs have been reported to mediate ADCC and antibody-dependent cellular virus inhibition (ADCVI) of the infected cells [55,74–76]. Antibodies that mediate ADCC and/or ADCVI activity(s) use their Fab region to bind to the epitope on gp120 or gp41 present on the surface of infected cells or on the virus attached to the infecting cells [55,74,76]. Meanwhile their Fc region binds to the Fc receptors (FcRs) on the effector cell which subsequently triggers cytotoxicity/cytolysis of the infected cells (ADCC) and/or non-cytotoxic antiviral activity(s) in the infected cell (ADCVI). The ADCC effector cells release cytolytic and cytotoxic molecules such as perforin and granzymes, respectively, whereas those with ADCVI activity produce chemokines (e.g., β chemokines) and/or cytokines that inhibit viral replication in the cell. The selective mutation(s) of the Fc region to remove FcR binding capability of the ADCC/ADCVI NAbs or ADCC/ADCVI nNAbs will result in the loss of ADCC and/or ADCVI activity(s). For example in an *in vitro* study, Fc mutated variants of wildtype (wt) ADCC-mediating bNAb (b12) retained potent viral neutralization activity similar to wt bNAb but lost ADCC activity [77]. However, a group of macaques passively immunized with wt bNAb b12 showed significant passive protection against SHIV challenge [78]. In comparison, the group passively immunized with Fc-mutant variant of wt b12 with diminished FcR binding potential had a significant loss in passive protection. The authors concluded that both bNAb activity and Fc-mediated activity(s) (ADCC, ADCVI) have synergist or additive effect on the protection against SHIV challenge.

NK cells, macrophages, dendritic cells, and neutrophils are the effector cells with FcRIIIa (CD16a) to mediate IgG-based ADCC activity [55,76]. ADCC antibodies target either linear or conformational epitopes on gp120 and gp41 [74]. In the RV144 trial, the nNAbs to the epitopes on V1V2 and C1 protein regions possessed ADCC activity which correlated with the modest protection observed in the vaccinated/protected subjects [7–9]. More specifically, the anti-V1V2 nNAbs with IgG3 subclass directly correlated with protection [8]. Although gp120 and gp41 are the predominant targets for ADCC antibodies [74], few studies have reported ADCC nNAbs to non-Env epitopes such as those on HIV Pol [79], Nef [80], and Vpu [81]. Nef [82–84] and part of Vpu [81,85] were reported to be present on the surface of HIV-infected cells, but such studies have not been reported for Pol [79]. Furthermore, serum from infected individuals showed a strong ADCC activity against a highly conserved, surface accessible linear Nef epitope (FLKEKGGLE) [80,84]. Overall, more studies will be needed to determine the importance of ADCC nNAbs to these HIV non-Env proteins.

Some nNAbs have been reported to enhance HIV infection by complement-mediated enhancement [86,87] or by FcR-mediated infection of dendritic cells and macrophages [33,88]. Whereas others may increase transcytosis of HIV-antibody IgG complex using FcR and DC-SIGN across the cell to present the HIV to the susceptible cells such as CD4+ T cells [89,90]. The binding of HIV-antibody complex to neonatal FcR (FcRn) on vaginal epithelial cells has been shown to enhance the transcytosis of HIV at low pH at the endosomal compartment [91], and these authors proposed that the FcRn detected in the genital tract may enhance the sexual transmission of HIV. In the RV144 trial, Env-specific blocking IgA nNAbs reduced the ADCC activity of the Env-specific IgG nNAbs by competing for the same epitope(s) [7,10]. Hence, an effective HIV vaccine should not induce HIV Env-specific blocking antibodies that will decrease the anti-HIV effects of ADCC and ADCVI antibodies or will decrease viral neutralization activity of the type-specific NAbs and bNAbs against HIV. The existence of enhancing and blocking Env-specific nNAbs suggests that a careful selection of protective B-cell epitopes on HIV Env may be needed for an effective HIV vaccine.

## Conserved T-cell epitopes associated with anti-HIV activity(s)

Conserved HIV T-cell epitopes for an effective HIV vaccine should induce broad (multiple subtype specificities) and potent (high magnitude) immunity against HIV. Conserved epitopes are often subdominant epitopes since excessive immunity against them or mutations will affect the fitness of the virus [92,93]. In addition, the immune responses to the dominant non-protective epitopes could potentially mask the immune responses to the protective conserved epitopes in a vaccinated host. Therefore, a vaccine consisting of only protective conserved epitopes may be ideal for an effective prophylaxis. During early HIV infection, ART-naïve HIV+ subjects initially produced CD8+ T-cell responses to predominantly variable epitopes than to conserved epitopes [94]. Conserved epitopes were identified more predominantly on Gag and Pol than on Env, Nef and accessory proteins [94–97]. Those HIV+ individuals who controlled HIV infection possessed CD8+ T-cell responses to conserved epitopes on Gag but not to those present on Pol [94]. Moreover, CD8+ T-cell responses to multiple conserved epitopes correlated with lower viral load set point. However, only a trend in lower viral load set point was observed in individuals possessing favorable HLA class-I alleles (e.g., HLA-B*27, HLA-B*57 [98]). Individuals who possess favorable HLA alleles are reported to undergo slow HIV/AIDS disease progression [98,99].

The conserved T-cell epitopes must be able to induce potent CD8+ CTL [34,35], polyfunctional CD8+ and CD4+ T-cell [100,101], and possibly CD4+ CTL [102] activities against HIV. These anti-HIV T-cell activities are considered to be important for vaccine prophylaxis based on the findings from HIV+ long-term non-progressor (LTNP) and elite controllers [103,104], RV144 trial [101], and NHP studies [105,106]. Less than 1% of the HIV+ subjects are elite controllers who have <50 copies/mL of circulating HIV, normal CD4+ T-cell counts, and are clinically asymptomatic [107,108]. Elite controllers maintain potent CD8+ CTL and polyfunctional T-cell responses against HIV [107,109] but no bNAbs and, if present, only a slight level of NAbs which alone cannot explain the undetectable levels of circulating HIV [110]. Polyfunctional T-cell responses include the production of cytokines and chemokines that inhibit HIV infection [22,111]. In particular, CD8+ CTLs in elite controllers produce higher levels of IL-2 and co-secretion of IL-2 and IFNγ but possess minimal breadth and level of IFNγ alone [22,110]. LTNPs also generate CD8+ CTLs [25] and non-lytic CD8+ T-cell antiviral factors (CAF) for the suppression of HIV replication [112].

In the modestly successful RV144 trial, the polyfunctional T-cell responses consisted of antigen-specific upregulation of IFNγ and IL-2 followed by TNF and IL-21 [101]. Some of these polyfunctional T cells also expressed CD107a, a marker for degranulation typically on CTLs. Furthermore, biological CTL analysis demonstrated the presence of anti-Env(V2) CD4+ CTLs. Notably, no CD8+ CTL activity was detected against HIV, whereas an earlier study using the same vaccine regimen showed CD8+ CTL activity against Env and Gag/Pol in 24% of the vaccines [113]. Recently, individuals with acute HIV infection were reported to have HIV-specific CD4+ CTLs with perforin/IFNγ or GrzA/IFNγ co-expression [102]. This study associates the early presence of CD4+ CTLs with slow disease progression.

A recent study demonstrated the importance of a vaccine capable of inducing CD8+ CTLs against a simian AIDS lentivirus, SIV. In this study, all macaques immunized with cytomegalovirus (CMV)-vectored SIV vaccine were positive for SIV infection shortly after the challenge with homologous SIV [105,106]. However, 50% of the vaccinated/infected macaques developed a transient infection which was completely cleared by CD8+ CTLs against SIV. This study demonstrates that at the early stage of infection, CD8+ CTLs can destroy and eliminate virus-infected cells. Moreover, this study establishes the importance of a vaccine inducing CD8+ CTLs for prophylaxis and immunotherapy.

## Conserved T-cell epitopes associated with protective HLA allotypes prevalent in HIV endemic countries

The T-cell epitopes bound to HLA class I and II molecules are recognized by the TCRs on CD8+ and CD4+ T cells, respectively to exert their function(s). Certain HLA allotypes (i.e., proteins expressed by HLA allele) confer resistance to HIV infection, while certain others increase susceptibility to the HIV infection [114–117]. HIV-resistant and -susceptible alleles may differ according to the race of the subjects and the circulating HIV subtype(s) prevailing in the endemic country. Alleles of HLA-A and HLA-B have been further classified into supertypes based on the similarity in their structural motif and their pocket chemical specificity to the peptides [118]. HLA alleles of supertype A2 such as HLA-A2, HLA-A*0205, HLA-A*6802 are associated with resistance to HIV subtype B in Caucasians from Europe and North America [119] and to the HIV subtypes A, C, and D in African population from Kenya and Tanzania of Sub-Sahara Africa (Table 3) [114–116,120]. Conversely, HLA alleles of supertype B7 such HLA-B*3501, HLA-B*3502 and HLA-B*5303 are associated with increased susceptibility to subtype-B HIV in Europe and North America [119], whereas supertype B7 alleles such as HLA-B*0702 and HLA-B*4201 are associated with increased susceptibility to HIV subtypes A, C, and D circulating in Kenya [117]. These alleles are not the only ones associated with increased susceptibility in Sub-Sahara Africa. HLA-A*2301 (supertype A24) in Kenya [114,117] and HLA-C*0702 in Tanzania [116] are also associated with increased susceptibility to HIV transmission.

Additionally, certain HLA allotypes correlate with slow disease progression, while certain others correlate with a rapid progression of the disease as determined by virus load, CD4+ T-cell count, and/or disease status [98,99]. HLA alleles associated with slow disease progression in Europe, North America, and Sub-Sahara Africa are members of supertype A3 (HLA-A*74, HLA-A*7401), supertype B27 (HLA-B*14), and supertype B58 (HLA-B*57, HLA-B*5703) and few other alleles (HLA-B*8101, C*1203, C*18, C*1801) (Table 4) [116,117,121–127]. Interestingly, these alleles are not the same ones associated with resistance to HIV. Similar to HLA alleles associated with elevated susceptibility to HIV, alleles in HLA supertype B7 are found in individuals with rapid HIV-disease progression, but the specific alleles are not always the same between the HIV-susceptible and the rapid HIV progression groups. In fact, the HLA alleles related with rapid HIV progression commonly found in Europe, North America, and Sub-Sahara Africa are HLA-B*07, B*0702, B*3501, B*3502, B*3503, and B*5301 of HLA supertype B7, and HLA-B*08 and HLAB-0801 of HLA supertype B8 [122,128–130] (Table 4). HLA-B*8101 which belongs to supertype B7 presents an anomaly to the trend of unfavorable alleles belonging to HLA supertype B7. HLA-B*8101 is associated with resistance to HIV infection in African Americans of North America [122] and Africans of Sub-Sahara Africa (South Africa, Botswana, Zimbabwe, Zambia) [125,127].

The HIV transmission studies evaluating resistant *versus* susceptible HLA alleles may help identify HIV epitopes and their corresponding immunity required for prophylaxis. In contrast, studies evaluating the control of the HIV infection in HIV+ subjects in terms of slow or rapid HIV progression may identify HIV epitope recognition and immunity more important for immunotherapy. In any event, the T-cell epitopes on HIV identified by these approaches should be tested for their ability to induce potent and broad anti-HIV T-cell activities.

## Antibodies and T-cell responses enhancing HIV infection

Non-protective T-cell epitopes can induce either neutral or enhancing effect on HIV infection. For instance, stimulation of CD4+ T cells can cause enhancement of HIV infection [131–133]. Autocrine and paracrine cytokine signaling, especially from TNF and IFNγ could increase in viral gene transcription simultaneously with the activation of CD4+ T-cells *via* NF-κB pathway [134–137]. Activated CD4+ T cells express high levels of HIV co-receptor CCR5 which in turn together with the primary receptor CD4 molecule will make the cell more susceptible to HIV infection [137]. The stimulation of CD4+ regulatory T cells (Treg) can have opposing effects on HIV infection. Treg cells can suppress anti-HIV CD8+ CTL activity which in turn will increase HIV infection [138], whereas Treg cells can suppress the production of inflammatory cytokines such as TNF and IFNγ which could decrease HIV viral replication by decreasing CD4+ T-cell activation [139,140]. In Step and Phambili trials, T cell-based (non-Env) vaccine caused enhancement of HIV infection (Tables 1 and 5) [12–14,21]. Furthermore, vaccine-induced enhancement upon SIV challenge was observed in NHPs vaccinated with SIV Env protein [141]. However, such enhancement was reported to be caused by CD4+ T-cell responses. In another animal AIDS model, FIV Env vaccination of cats enhanced challenge infection with FIV [142,143]. In the RV144 trial, Env-specific IgA antibodies decreased the ADCC activity of Env-specific IgG [5,8]. Therefore, an ideal vaccine should limit unwanted CD4+ T cell activation and non-specific cytokine production to minimize viral replication, whereas it should activate potent anti-HIV effector activities such those of anti-HIV CTL, anti-HIV polyfunctional T-cells, and bNAb/NAb/ADCC antibodies [132].

## Conclusion

### Conserved T-cell and B-cell epitopes with potent anti-HIV activity for HIV vaccine

As discussed above, significant progress has been made to identify the protective B-cell and T-cell epitopes for a highly effective HIV vaccine. Such HIV vaccine should consist predominantly of anti-HIV conserved T-cell epitopes (Table 5B) and selected B-cell epitopes that induce bNAbs, NAbs, and ADCC/ADCVI antibodies (Table 5A) with exclusion of HIV-enhancing epitopes. To this end, studies are in progress using HIV mosaic vaccines consisting of anti-HIV conserved T-cell epitopes and epitope regions rather than whole HIV proteins which may contain enhancing epitopes [45,46,144–147]. The development of conformational B-cell epitopes that induce bNAbs appears to be a major task since Env vaccine epitopes for bNAbs are still unavailable [31,34,56,57]. Concerted efforts have been made in developing Env trimers. However, current whole-Env trimer constructs still contain HIV-enhancing epitopes such as epitopes for blocking antibodies and T-cell epitopes that stimulate immunodominant responses without anti-HIV activity(s) while masking the subdominant protective responses. Consequently, if the trimer immunogen does not induce potent bNAbs as well as potent NAbs to multiple subtypes then HIV-enhancing epitopes and non-protective HIV immunodominant epitopes can counteract the potency of these antibodies, and the vaccine efficacy may not be detected such as those observed in VAX003 and VAX004 trials. Hence, the selection of minimally-constructed protective epitopes (without enhancing epitopes) is required.

Until minimally constructed, highly potent bNAb conformational epitopes become available, the use of bNAb linear epitopes (e.g., MPER) and most potent NAb linear epitopes from multiple subtypes (A,B,C) should be combined as mosaic vaccine and tested in SHIV/macaque model. Finally, besides bNAbs and type-specific NAbs, every effort should be made to include other conserved B-cell epitopes such as those that induce potent and broad ADCC/ADCVI antibodies as well as conserved T-cell epitopes that induce potent and broad anti-HIV polyfunctional T-cell responses and anti-HIV CD8+ and CD4+ CTLs. T cell-based conserved-mosaic vaccines were shown to enhance the breadth and potency of epitope recognition [147]. In another study, short conserved HIV epitopes devoid of immunodominant epitopes were shown to increase immunogenicity and to shift the immunodominance [148]. The RV144 trial is considered to be the first effort to combine B-cell and anti-HIV cellular immune effector activities (i.e., T cells and NK cells). More importantly, this trial is the only phase-III trial that demonstrated prophylactic efficacy although of modest level. In fact, the findings from this trial further support the contention that more anti-HIV immune effector activities are required to confer complete or sterilizing protection. In conclusion, an efficacious HIV vaccine may need to stimulate multitude of potent and broad immune effector activities against HIV in order to confer sterilizing protection against one of this century’s most challenging viral pathogen, HIV.

## Figures and Tables

**Table 1:** Selected prophylactic HIV vaccine trials with major contributions toward identifying vaccine epitopes and/or immunity.

Vaccine Trial	Vaccine Immunogen	Efficacy [No. infected]	Vaccine-induced Immune Responses
**B-cell Based Vaccine**			
Phase-III VAX003 (Thailand)	AIDSVAX gp120 B/E Subtype-B MN & CRF01_AE	No efficacy [4] [83/1017 vaccine vs. 81/1013 placebo]	Binding nNAbs and tier-1 NAbs to gp120 (sporadic weak bNAb) [4,149] IgG4 bias is associated with reduced Ab-mediated Fc-effector function [150]
Phase-III VAX004 (North America & Netherland)	AIDSVAX gp120 B/B Subtype-B MN & GNE8	No efficacy [5] [241/3598 vaccine vs. 127/1805 placebo]	Binding nNAbs and NAbs to gp120 (no bNAb) [151] ADCC Abs are associated with lower infection risk [152] CD8+ T-cell proliferation significantly higher in HIV infected group than uninfected group [153]
**B-cell and T-cell Based Vaccine**			
Phase-III RV144 (Thailand)	Priming vaccine: ALVAC-gag-pro-env Subtype-B LAI gag & pro CRF01_AE gp120 Subtype-B LAI gp41* (*no ectodomain) Boosting vaccine: AIDSVAX gp120 B/E	Positive efficacy [6] All risk groups 31.2% [51/7960 vaccine vs. 74/7988 placebo] High risk group 3.7% [22/1896 vaccine vs. 23/1929 placebo]	IgG to V1V2 has significant inverse correlation with infection [7] Env-specific IgA Abs have significant direct correlation with infection [7] ADCC and Env-specific CD4+ T cells correlate inversely with infection [7] Tier-1 NAbs lower in peak tier than those in Vax003 and no bNAbs [147] IgG3 to V1V2 correlate with reduced infection risk [8] IgG to V2 & V3 linear epitopes correlate with reduced infection risk [9] IgA Abs to C1 block binding and ADCC effector function of IgG [10] ADCVI-like activity correlate directly with IgG1 & IgG2 to gp120 [150] Presence of Env V2-specific polyfunctional CD4+ T-cell responses of IFNγ and IL2 followed by TNFα and then IL21 [101] Presence of Env V2-specific CD4+ CTLs [101] No HIV-specific CD8+ T-cell (ICS) responses to Gag or Env [6]
**T-cell Based Vaccine**			
Phase-IIb HVTN 502 or Step (North & South America, Caribbean, Australia)	Ad5-gag/pol/nef Subtype B	Negative efficacy [12] [Enhanced infection: 24/741 vaccine vs. 21/762 placebo]	Nonspecific IFNγ secretion, but not HIV-specific IFNγ, is associated with increased HIV infection risk [21] 43% vaccinees with HIV-specific CD8+ T-cell responses [23] Low in breadth compared to SIV vaccine studies Low in magnitude compared to LTNP with same assay More HIV-specific IFNγ alone or IFNγ/TNFα than IL2 Small percentage of vaccinees express IL2 41% vaccinees with HIV-specific CD4+ T-cell responses [23] 31% vaccinees with HIV-specific CD4+ and CD8+ T-cell responses after all vaccinations [23] Pre-existing anti-Ad5 Abs reduce HIV-specific productions of IFNγ, IL2, or both more profoundly in CD8+ T cells than in the CD4+ T cells [23]
Phase-IIb HVTN 503 or Phambili (South Africa, >98% Black population)	Ad5-gag/pol/nef Subtype B	Negative efficacy [14] [9 mos: 34/400 vaccine vs. 28/400 placebo [13]; enhanced in 42 mos: 63/400 vaccine vs. 37/400 placebo [14]]	More vaccinees with IFNγ-secreting T-cell responses to Gag & Nef from subtype B than from subtype C with the exception of Pol [13] Higher IFNγ titers to subtype-B Pol and Nef than to those of subtype C; similar IFNγ titers in response to subtype-B and -C Gags [13] 53% vaccinees with IFNγ responses to all 3 subtype-B antigens but 15% vaccinees with responses to all 3 subtype-C antigens [13]

**Table 2:** B-cell epitope specificity of bNAbs.

Env Target Specificity	bNAb ID	Discontinuous or Linear Epitope	Ab Isotype	ADCC Activity^a^	ADCC Reference^a^
CD4bs	VRCO1	Discontinuous	IgG1	ADCC	[154,155]
	3BNC117	Discontinuous	IgG1κ	ADCC	[154,156,157]
	CH103	Discontinuous	IgG1	na	na
	b12	Discontinuous	IgG1	ADCC	[158,159]
V2 Proteoglycan	PG9	Discontinuous	IgG1	ADCC	[154,156,157]
	CHO1	Discontinuous	IgG1	na	na
	PGT145	Discontinuous	IgG	ADCC	[156]
	VRC2609	Discontinuous	IgG	na	na
V3 Proteoglycan	PGT121	Discontinuous	IgG1	ADCC	[154,156]
	PGT128	Discontinuous	IgG1	na	na
	PGT135	Discontinuous	IgG	na	na
MPER	10E8	Linear	IgG3	ADCC	[154]
	4E10	Linear	IgG3κ	ADCC	[156,159]
	2F5	Linear	IgG3	ADCC	[156,159,160]
gp120-gp41	PGT151	Discontinuous	IgG	Negative	[161]
	VRC34.01	Discontinuous	IgG	na	na
	35022	Discontinuous	IgG	ADCC	[157]
	8ANC195	Discontinuous	IgG	Negative	[161]

a Not available (na)

**Table 3:** HLA class-I alleles associaed with resistance or susceptible to HIV infection.

HLA Class I	HLA Supertype^a^	HIV Subtype^b^	Cohort Size	Cohort & Study Description^c^	Reference
**Europe & North America: HIV-Resistant HLA**					
A*02/A*0205/A*6802	A2	B	284	Caucasian homosexual transmission	[119]
Sub-Sahara Africa: HIV-Resistant HLA					
A*02/A*6802	A2	A,D,C	433	Kenya; M-C & perinatal transmission	[120]
		A,D,C	232	Kenya; CSW heterosexual transmission	[114]
		A,D,C	171	Kenya; M-C & perinatal transmission	[115]
A*0205	A2	A,C,D	272	Tanzania; seroconversion survey	[116]
**Europe & North America: HIV-Susceptible HLA**					
B*3501/B*3502/B*3503	B7	B	284	Caucasian homosexual transmission	[119]
**Sub-Sahara Africa: HIV-Susceptible HLA**					
A*2301	A24	A,D,C	232	Kenya; CSW heterosexual transmission	[114]
		A,D,C	338	Kenya; CSW heterosexual transmission	[117]
B*0702	B7	A,D,C	338	Kenya; CSW heterosexual transmission	[117]
B*4201	B7	A,D,C	338	Kenya; CSW heterosexual transmission	[117]
C*0702	C*07	A,C,D	272	Tanzania; seroconversion survey	[116]

a Two-digit resolution nomenclature for HLA-C.

b HIV-1 subtypes shown in order of prevalence and based on following references [162–166].

c Black populations from Sub-Sahara; mother-to-child (M-C) transmission; female commercial sex worker (CSW)

**Table 4:** HLA class-I alleles associated with HIV disease progression.

HLA Class I	HLA Supertype^a^	HIV Subtype^b^	Cohort Size	Cohort Description	Reference
**Europe & North America: HLA for HIV Slow Progression**					
A*74/A*7401	A3	B	338	African American	[123]
B*14	B27	B	2,945	Caucasian & African American	[122]
		B	338	African American	[123]
B*57	B58	B	338	African American	[123]
		B	241	Caucasian	[121]
		B	2,945	Caucasian, African American	[122]
B*5703	B58	B	3,622	African American, Hispanic^c^	[122]
		B	338	African American	[123]
B*8101	B7	B	2,945	African American^c^	[122]
C*1203	C*12	B	2,945	Caucasian, African American	[122]
C*18/C*1801	C*18	B	2,945	African American^c^	[122]
		B	338	African American	[123]
**Sub-Sahara Africa: HLA for HIV Slow Progression**					
A*74/A*7401	A3	C	784	Zambia	[125]
		A,C,D	508	Tanzania	[116]
		A,D,C	663	Kenya	[117]
B*14	B27	A,D,C	663	Kenya	[117]
B*57	B58	C	259	Zambia	[124]
B*5703	B58	C	1,211	South Africa	[126]
		C	784	Zambia	[125]
		C	2,126	South Africa, Botswana, Zimbabwe	[127]
		A,D,C	663	Kenya	[117]
		A,C,D	329	Tanzania (only females)	[116]
B*8101	B7	C	563	Zambia	[125]
		C	2,126	South Africa, Botswana, Zimbabwe	[127]
C*1203	C*12	C	2,216	South Africa, Botswana, Zimbabwe	[127]
C*18	C*18	C	784	Zambia	[125]
C*1801	C*18	A,C,D	329	Tanzania (only females)	[116]
**Europe & North America: HLA for HIV Rapid Progression**					
B*07/B*0702	B7	B	2,945	Caucasian^d^	[122]
B*3501	B7	B	2,945	Caucasian, African American	[122]
		B	1,089	Caucasian, African American, Hispanic	[130]
B*3502/B*3503	B7	B	850	Caucasian, African American	[129]
		B	1,089	Caucasian, African American	[130]
B*5301	B7	B	850	Caucasian, African American	[129]
		B	2,945	Caucasian	[122]
		B	1,089	Caucasian, African American, Hispanic	[130]
B*08/B*0801	B8	B	32	Caucasian	[128]
		B	2,945	Caucasian^d^	[122]
**Sub-Sahara Africa: HLA for HIV Rapid Progression**					
B*07/B*0702	B7	A,D,C	663	Kenya	[117]
B*3501	B7	C	2,126	South Africa, Botswana, Zimbabwe	[127]
B*3502/B*3503	B7	A,D,C	663	Kenya	[117]
B*5301	B7	A,D,C	663	Kenya	[117]
B*08/B*0801	B8	C	2,126	South Africa, Botswana, Zimbabwe	[127]

a Two-digit resolution nomenclature for HLA-C.

b HIV-1 subtypes shown in order of prevalence and based on following references [163–165].

c Not in Caucasian population.

d Not in African American population.

**Table 5:** Features of B- and T-cell epitopes for an efficacious HIV vaccine.

**A. B-cell epitopes for prophylactic vaccine:**
· Generate potent bNAbs
· Generate bNAbs of known Env targets (e.g., linear epitope for 10E8) until more potent minimally constructed bNAb epitopes become available
· Generate potent type-specific NAbs to the subtype(s) prevalent in the country
· Generate broad and potent ADCC/ADCVI epitopes
· Must exclude HIV epitopes that induce neutralization-blocking Abs and HIV infection-enhancing Abs
**B. T-cell epitopes for prophylactic vaccine:**
· Should be highly conserved to prevent the development of escape mutants
· Generate broad and potent anti-HIV CD8^+^ CTLs secreting perforin and/or granzymes
· Generate broad and potent polyfunctional T-cell responses especially those that induce IL2/proliferation and anti-HIV β-chemokines
· Possibly induce CD4+ CTLs without activation of HIV-susceptible CD4^+^ T cells
· Should recognize HLA allotypes prevalent in the countries
· Must exclude HIV-infection enhancing epitopes
